# Why are biosimilars much more complex than generics?

**DOI:** 10.31744/einstein_journal/2019ED4836

**Published:** 2019-02-19

**Authors:** Eduardo Pagani

**Affiliations:** 1 Laboratório Nacional de Biociências, Centro Nacional de Pesquisas em Energia e Materiais, Campinas, SP, Brazil.

The regulatory demands for registering a generic medicine are physical-chemical tests and one comparative oral bioavailability clinical trial (bioequivalence).^(^ ^1^ ^)^ This takes about 2 years, costs between USD$1 million and 2 million and enables claims for all indications accepted for the original drug. Demands for a biosimilar include many expensive and time-consuming comparative tests, and efficacy clinical trials for each indication. This takes from 5 to 9 years and costs over USD$100 million.^(^ ^2^ ^)^

The concept of generic medicine was developed for oral drugs, and biologics are mostly injectable. Moreover, the reasons for this huge difference are molecular complexity and the manufacturing process. The active pharmaceutical ingredients (APIs) of generics are small molecules, with a molecular weight of approximately 500 Daltons. Generic API purity and identity are mostly evaluated by inexpensive chromatographic tests. A bioequivalence clinical trial assesses the pharmacokinetic impact of the formulation and manufacturing of the API.^(^ ^3^ ^)^ Since the APIs are the same, have the same concentration and a comparable bioavailability, regulatory authorities assume that the clinical effects will be the same for all indications.

Most biologic APIs are proteins produced by cell culture. They are big, complex and variable. The molecular weight of a monoclonal antibody exceeds 150,000 Daltons. Its complex structure is organized in four levels. Glycosylation is very important for function and it is not identical for all molecules of the same batch. Finally, proteins form aggregates of different sizes. Therefore, a batch of biologic API is a pool, rather than of a lot of identical molecules. There are also impurities from the cells, culture media and the purification process that can be biologically active, which adds even more complexity.

Therefore, each biological product, be it original or biosimilar, is unique and has individual characteristics that come from the manufacturing process. The saying “the process is the product” summarizes the thought in this field. Some products even have specific regulations that might differ from one country to another.

Biotechnology evolves fast. New technologies are much more productive than those available when a certain original drug was created. Therefore, the regulatory challenge is to reach a conclusion about the clinical interchangeability of therapeutic protein polls manufactured in different plants, by different processes.^(^ ^4^ ^-^ ^6^ ^)^

That is why regulatory demands are so extensive. Almost all tests demanded for an innovative biologic drug shall be made in comparison to the reference drug for a biosimilar.^(^ ^7^ ^,^ ^8^ ^)^ This is called comparability exercise, and includes physical-chemical assessments, functional assays, animal tests, and clinical trials.

The comparability exercise can be more complex than simply meeting the regulatory demands for an innovative biologic. High costs and a high risk of failure are barriers for some Brazilian industries to develop biosimilars. However, this is part of the definitive solution for Brazilian independence in this very important pharmaceutical area, and for a radical innovation.

One method to reduce risk is to assure the best possible API similarity from the very beginning. This means assuring that two protein populations, produced in different plants, by different processes and different equipment are similar enough to justify further investments. The goal is that the differences between a biosimilar and its reference biologic be small enough not to be clinically significant. Modern techniques of protein structure, such as crystallography with synchrotron light, mass spectrometry, spectroscopy and *in vitro* functional assays are the light at the end of the tunnel.
